# Construction and validation of coronary heart disease risk prediction model for general hospitals in Tacheng Prefecture, Xinjiang, China

**DOI:** 10.3389/fcvm.2024.1514103

**Published:** 2024-12-12

**Authors:** Yikang Xu, Jingru Ma, Yang Yang, Limin Liu, Xinran Zhao, Yu Wang, Alimu Mijiti, Qiangru Cheng, Jun Ma

**Affiliations:** ^1^ Department of Cardiovascular Medicine, The Second Affiliated Hospital of Shenyang Medical College, Shenyang, China; ^2^ School of Public Heath, Shenyang Medical College, Shenyang, China; ^3^ Department of Cardiovascular Medicine, Tacheng People’s Hospital, Tacheng, China

**Keywords:** Tacheng Prefecture, Xinjiang, coronary heart disease, nomograms, risk prediction, logistic regression

## Abstract

**Objective:**

To analyze the risk factors for coronary heart disease (CHD) in patients hospitalized in general hospitals in the Tacheng Prefecture, Xinjiang, and to construct and verify the nomogram prediction model for the risk of CHD.

**Methods:**

From June 2022 to June 2023, 489 CHD patients (CHD group) and 520 non-CHD individuals (control group) in Tacheng, Xinjiang, were retrospectively selected. Using a 7:3 ratio, patients were divided into a training group (706 cases) and a validation group (303 cases). General clinical data were compared, and key variables were screened using logistic regression (AIC). A CHD risk nomogram for Tacheng was constructed. Model performance was assessed using ROC AUC, calibration curves, and DCA.

**Results:**

In the training group, non-Han Chinese (OR = 2.93, 95% CI: 2.0–4.3), male (OR = 1.65, 95% CI: 1.0–2.7), alcohol consumption (OR = 1.82, 95% CI: 1.2–2.9), hyperlipidemia (OR = 2.41, 95% CI: 1.7–3.5), smoking (OR = 1.61, 95% CI: 1.0–2.6), diabetes mellitus (OR = 1.62, 95% CI: 1.1–2.4), stroke (OR = 2.39, 95% CI: 1.6–3.7), older age (OR = 1.08, 95% CI: 1.1–1.2), and larger waist circumference (OR = 1.04, 95% CI: 1.0–1.1) were the risk factors for coronary heart disease (all *P* < 0.05). The area under the curve (AUC) of the work characteristics of the subjects in the training group and the validation group were 0.80 (95% CI: 0.8–0.8) and 0.82 (95% CI: 0.8–0.9), respectively. The Hosmer-Lemeshow test indicated *P* = 0.325 for the training group and *P* = 0.130 for the validation group, with calibration curves closely fitting the ideal curve. The predicted values aligned well with actual values, and decision curve analysis results suggest that the model offers a net clinical benefit.

**Conclusion:**

The CHD risk prediction model developed in this study for general hospitals in Tacheng Prefecture, Xinjiang, demonstrates strong predictive performance and serves as a simple, user-friendly, cost-effective tool for medical personnel to identify high-risk groups for CHD.

## Introduction

According to data from the Global Coronary Heart Disease (CHD) Burden Report from 1990 to 2019, about 197 million people worldwide suffered from CHD, and about 9.14 million died due to CHD. The age-standardized mortality rate of CHD increased from 99 per 100,000 to 116.4 per 100,000 (1, 2). “Analysis of the Mortality Trend of Ischemic Heart Disease in China from 2010 to 2019” shows that the prevalence of CHD in China will increase in the next decade, and its mortality rate and age-adjusted mortality rate will continue to increase, with an increase of 5.14% and 1.60%, respectively (3). According to the “China Cardiovascular Health and Disease Report 2022” (4), there are about 11.39 million patients with CHD in China (5). Studies have shown that the burden of ischemic heart disease in northern regions such as Xinjiang, Heilongjiang, and Jilin in China is four times that of southern areas (6). At the same time, according to the Chinese Cardiovascular Health Index, the distribution of cardiovascular disease health resources and disease treatment capacity in Northwest China are also relatively backward (7, 8). Tacheng area of Xinjiang is a multi-ethnic area, which has apparent differences in dietary characteristics and living habits from other groups and has a different emphasis on risk factors and prevention of CHD. Although some studies have analyzed the incidence of coronary heart disease and its risk factors in the Xinjiang region, there has been no study specifically focusing on the situation in the Tacheng region. Early identification of high-risk groups for CHD is crucial for reducing disease incidence and enhancing population health and quality of life. Numerous studies have shown that assessing and screening these groups to eliminate health risk behaviors can lower the prevalence of CHD (9–11).

Risk prediction models can assist in the formulation of the best preventive or therapeutic interventions. In recent years, risk prediction models have been most widely used in the primary prevention of CHD in China, among which the latest recommended models in Chinese guidelines (12, 13) include the CMCS model based on the Chinese Multi-provincial Cohort Study (CMCS) and China-PAR model based on China-PAR research. The CMCS model incorporates risk factors such as age, low-density lipoprotein cholesterol (LDL-C), systolic blood pressure, high-density lipoprotein cholesterol (HDL-C), diabetes mellitus, and smoking. In contrast, the China-PAR model includes a wider array of risk factors: sex, age, residence (urban or rural), geographic location (north or south of the Yangtze River), waist circumference, total cholesterol (TC), HDL-C levels, current blood pressure status, antihypertensive medication use, diabetes status, smoking habits, and family history of cardiovascular disease. Notably, ethnic distribution and regional variations are not considered in the CMCS model's formula. The China-PAR model categorizes regional risk factors based solely on north-south delineation by the Yangtze River without accounting for ethnic diversity. The Tacheng area of Xinjiang is characterized by its multi-ethnic population with distinct cultural attributes; dietary practices and lifestyle habits vary significantly among these groups. To date, no large-scale screening studies targeting high-risk cardiovascular populations have been conducted in Tacheng. Consequently, our understanding of these populations and their associated risk factor distributions remains limited. Furthermore, insufficient research has focused on identifying high-risk cardiovascular groups within this region to effectively inform early prevention strategies. As a result, efforts toward early screening and prevention of cardiovascular diseases in Tacheng have not achieved expected outcomes.

Therefore, this study explores the risk factors of CHD in patients hospitalized in general hospitals in the Tacheng area, constructs a risk prediction model using logistic regression, and presents it in the form of a nomogram to provide a scientific basis for the early identification of people at high risk of CHD, the formulation of precise preventive measures, and the delay of the occurrence of CHD.

## Materials and methods

### Ethics and consent

This study was approved by the Ethics Committee of our hospital (approval number: 2023-Ethics of Shen Medical Second Hospital—48; The Ethics Committee of Tacheng People's Hospital 2022-12-25). There is a clear statement that all the patients have written informed consent and consented to the analysis and publishing of all clinical data in the study.

### Research objects

From June 2022 to June 2023, 489 patients with CHD who underwent elective coronary angiography were collected from the cardiovascular departments of several general hospitals in the Tacheng area as the experimental group, all of whom were diagnosed with CHD according to the results of coronary arteriography, and 520 cases of healthy medical checkups from the physical examination centers of the hospitals were selected as the control group during the same period. Inclusion criteria: (1) age > 18 years; (2) diagnosis of CHD based on coronary angiography results, at least one major coronary artery stenosis greater than 50% (14). Exclusion criteria: ① severe heart valve disease, rheumatic heart disease, coagulation disorders, liver and renal failure, pulmonary failure, severe infections, immune system diseases, and malignant tumors, etc.; ② patients with missing case information. This study was approved by the Ethics Committee of our hospital (approval number: 2023-Ethics-48 of the Second Affiliated Hospital of Shenyang Medical College; The Ethics Committee of Tacheng People's Hospital 2022-12-25).

### Data collecting

The patient's social-demographic data, disease-related data, and various laboratory examination indicators were collected by consulting the electronic records. Social-demographic data included age, gender, ethnicity, smoking history, drinking history, BMI, waist circumference, marital status, heart rate, and blood pressure at admission. Disease-related data included hyperlipidemias, diabetes mellitus, hypertension, chronic renal insufficiency, atrial fibrillation/atrial flutter, history of heart failure, history of old myocardial infarction, and stroke. Laboratory data included triglycerides, cholesterol, high-density lipoprotein, low-density lipoprotein, atrial natriuretic factor, blood potassium, blood sodium, blood chlorine, serum creatinine, uric acid, blood urea nitrogen, hypersensitivity C-reactive protein, carotid color Doppler ultrasound and selective coronary angiography.

### Statistical analysis

Statistical analyses were conducted using SPSS version 25.0 and R software (version 4.0.5). Baseline data for both groups were presented as frequencies, percentages, and means ± standard deviations (SD). For intergroup comparisons, a *t*-test was employed for normally distributed continuous variables; in cases where the data did not conform to normal distribution, the rank sum test was utilized for continuous variables, while chi-square tests were applied to categorical data. A two-tailed *P*-value < 0.05 was considered statistically significant.

A total of 1,009 subjects were enrolled in this study and randomly assigned to a training group and a validation group in a 7:3 ratio using the random number table method. The training group employed logistic regression to develop a predictive model, which was subsequently presented as a nomogram, while the validation group served for internal validation purposes. Key variables for the model were identified based on the Akaike Information Criterion (AIC) through univariate and multivariate logistic two-way stepwise regression methods; specifically, variables with *P*-values < 0.05 from univariate analysis were incorporated into the multivariate logistic regression model. The predictive performance, calibration, and clinical utility of the model were evaluated using the area under the curve (AUC) of receiver operating characteristic (ROC) analysis, calibration curves, and decision curve analysis (DCA). A *P*-value threshold of <0.05 was considered statistically significant. An AUC value ranging from 0.5 to 0.7 indicates that the prediction performance of the model is moderate. The value of AUC is 0.7–0.9, meaning that the model has good prediction performance. When the AUC value is more significant than 0.9, the model's prediction performance is excellent (15).

## Results

### Basic information about the research subjects

One thousand nine subjects, 581 (57.6%) males and 235 (42.4%) females, aged (61.4 ± 11.6) years, were included in the present study and were randomly divided into a training group (706) and a validation group (303) in a ratio of 7:3 using a randomized numerical table. A comparison of the clinical data of the patients in the training and validation groups showed that the difference between the two groups was not statistically significant and was balanced (*P* > 0.05, Table 1).

**Table 1:** Comparative table of patients’ clinical data.

Variables	Total (*n* = 1,009)	Validation set (*n* = 303)	Training set (*n* = 706)	t/*χ*² value	*P* value
Age (year, mean ± SD)	61.4 ± 11.6	61.9 ± 12.3	61.1 ± 11.4	1.0	0.298
BMI (kg·m^−2^, mean ± SD)	27.1 ± 3.8	27.1 ± 3.9	27.0 ± 3.7	0.0	0.982
Waistline (cm, mean ± SD)	87.7 ± 10.2	88.1 ± 10.4	87.5 ± 10.1	0.9	0.389
Heart rate (times/min, mean ± SD)	76.5 ± 13.7	76.8 ± 13.6	76.5 ± 13.7	0.3	0.732
Systolic blood pressure (mmHg, mean ± SD)	137.0 ± 21.1	138.7 ± 21.6	136.2 ± 20.8	1.7	0.085
Diastolic blood pressure (mmHg, mean ± SD)	83.3 ± 13.0	83.6 ± 12.5	83.1 ± 13.2	0.6	0.573
TG (mmol/L, mean ± SD)	2.0 ± 0.4	2.0 ± 0.3	2.1 ± 0.4	−0.8	0.455
TC (mmol/L, mean ± SD)	4.8 ± 1.1	4.8 ± 1.1	4.8 ± 1.1	−0.1	0.926
HDL-C (mmol/L, mean ± SD)	1.2 ± 0.3	1.2 ± 0.3	1.2 ± 0.3	0.8	0.400
LDL-C (mmol/L, mean ± SD)	2.9 ± 0.8	2.8 ± 0.8	2.9 ± 0.8	−0.1	0.891
Blood potassium (mmol/L, mean ± SD)	3.9 ± 0.4	3.9 ± 0.4	3.9 ± 0.4	−0.4	0.690
Blood sodium (mmol/L, mean ± SD)	140.8 ± 2.6	140.8 ± 2.6	140.8 ± 2.7	0.3	0.780
Blood chlorine (mmol/L, mean ± SD)	104.3 ± 2.9	104.3 ± 3.0	104.3 ± 2.9	0.4	0.673
Creatinine (umol/L, mean ± SD)	71.2 ± 25.7	72.2 ± 24.9	70.8 ± 26.0	0.7	0.457
Uric acid (umol/L, mean ± SD)	330.3 ± 108.0	335.8 ± 108.2	327.9 ± 108.0	1.1	0.284
Urea nitrogen (mmol/L, mean ± SD)	6.0 ± 2.2	6.1 ± 2.2	6.0 ± 2.2	0.9	0.355
Hs-CRP (mmol/L, mean ± SD)	14.5 ± 4.1	15.5 ± 4.3	14.1 ± 3.9	1.4	0.178
Gender *n* (%)				0.1	0.832
Female	428 (42.4)	127 (41.9)	301 (42.6)		
Male	581 (57.6)	176 (58.1)	405 (57.4)		
Race *n* (%)				0.0	0.925
Han Chinese	414 (41.0)	125 (41.3)	289 (40.9)		
Non-Han	595 (59.0)	178 (58.8)	417 (59.1)		
Drinking *n* (%)				0.6	0.441
No	638 (63.2)	197 (65.0)	441 (62.5)		
Yes	371 (36.8)	106 (35.0)	265 (37.5)		
Hyperlipidemia *n* (%)				0.2	0.656
No	467 (46.3)	137 (45.2)	330 (46.7)		
Yes	542 (53.7)	166 (54.8)	376 (53.3)		
Marital status *n* (%)				0.3	0.603
Married	891 (88.3)	270 (89.1)	621 (88.0)		
Other marriages	118 (11.7)	33 (10.9)	85 (12.0)		
CVD family history, *n* (%)				3.5	0.060
No	711 (70.5)	226 (74.6)	485 (68.7)		
Yes	298 (29.5)	77 (25.4)	221 (31.3)		
Smoking *n* (%)				0.3	0.568
No	603 (59.8)	177 (58.4)	426 (60.3)		
Yes	406 (40.2)	126 (41.6)	280 (39.7)		
Carotidplaque *n* (%)				1.1	0.300
No	511 (50.6)	161 (53.1)	350 (49.6)		
Yes	498 (49.4)	142 (46.9)	356 (50.4)		
Diabetes *n* (%)				1.7	0.197
No	721 (71.5)	225 (74.3)	496 (70.3)		
Yes	288 (28.5)	78 (25.7)	210 (29.8)		
Hypertension *n* (%)				0.0	0.881
No	293 (29.0)	87 (28.7)	206 (29.2)		
Yes	716 (71.0)	216 (71.3)	500 (70.8)		
Chronic renal insufficiency *n* (%)				0.1	0.789
No	963 (95.4)	290 (95.7)	673 (95.3)		
Yes	46 (4.6)	13 (4.3)	33 (4.7)		
Atrial fibrillation/atrial flutter *n* (%)				1.2	0.266
No	946 (93.8)	288 (95.1)	658 (93.2)		
Yes	63 (6.2)	15 (5.0)	48 (6.8)		
Heart failure *n* (%)				1.2	0.283
No	767 (76.0)	237 (78.2)	530 (75.1)		
Yes	242 (24.0)	66 (21.8)	176 (24.9)		
History of stroke *n* (%)				3.0	0.083
No	727 (72.1)	207 (68.3)	520 (73.7)		
Yes	282 (28.0)	96 (31.7)	186 (26.4)		

### Screening of risk factors for coronary heart disease

In the training group, single-factor logistic regression analysis showed that the differences in the occurrence of CHD between whether or not they were Han Chinese, different genders, whether or not they consumed alcohol, whether or not they were hyperlipidemic, whether or not they smoked, whether or not they were diabetic, whether or not they were chronically renal insufficient, whether or not they had heart failure, whether or not they had a stroke, different ages, different waist circumferences, different levels of systolic blood pressure at the time of admission, and different levels of uric acid had statistically significant effects on the occurrence of CHD (*P* < 0.05, Table 2). The screening of nomogram predictors was based on the AIC criterion using a multifactorial logistic two-way stepwise regression method. The final variables that were included in the nomogram predictive model were: non-Han ethnicity (OR = 2.93, 95% CI: 2.0–4.3, *P* < 0.001), males (OR = 1.65, 95% CI: 1.0–2.7, *P* = 0.040), alcohol consumption (OR = 1.82, 95% CI: 1.2–2.9, *P* = 0.009), hyperlipidemia (OR = 2.41,95% CI: 1.67–3.49, *P* < 0.001), smoking (OR = 1.61,95% CI: 1.01–2.55, *P* = 0.045), diabetes (OR = 1.62, 95% CI: 1.1–2.4 *P* = 0.015), stroke (OR = 2.39, 95% CI: 1.6–3.7, *P* < 0.001), older age (OR = 1.08, 95% CI: 1.1–1.1, *P* < 0.001), and larger waist circumference (OR = 1.04, 95% CI: 1.0–1.1, *P* < 0.001) were the risk factors for CHD (Table 3).

**Table 2:** Results of single-factor regression analysis of coronary heart disease risk factors in the training group.

	*β*	S.E	Z	*P*	OR (95% CI)
Race	1.12	0.2	7.0	**<**.**001**	3.06 (2.24–4.18)
Gender	0.62	0.2	4.0	**<**.**001**	1.86 (1.37–2.53)
Drinking	0.75	0.2	4.8	**<**.**001**	2.12 (1.55–2.88)
Hyperlipidemia	0.89	0.2	5.8	**<**.**001**	2.44 (1.80–3.31)
Smoking	0.64	0.2	4.1	**<**.**001**	1.89 (1.40–2.56)
Diabetes	0.63	0.2	3.7	**<**.**001**	1.87 (1.34–2.61)
Chronic renal insufficiency	0.88	0.3	2.4	**0**.**018**	2.41 (1.16–4.99)
Heart failure	0.73	0.2	4.1	**<**.**001**	2.08 (1.46–2.96)
Stroke	1.05	0.2	5.8	**<**.**001**	2.86 (2.01–4.07)
Age	0.04	0.0	5.8	**<**.**001**	1.04 (1.03–1.05)
Waistline	0.05	0.0	5.7	**<**.**001**	1.05 (1.03–1.07)
Systolic blood pressure	0.01	0.0	2.3	**0**.**023**	1.01 (1.01–1.02)
Uric acid	0.01	0.0	2.1	**0**.**033**	1.01 (1.01–1.01)

Bold typeface indicates statistically significant differences where the *P* value is less than 0.05.

**Table 3:** Results of multifactorial stepwise regression analysis of coronary heart disease risk factors in the training group.

Variables	*β*	S.E	Z	*P*	OR (95% CI)
Race	1.07	0.19	5.6	**<**.**001**	2.93 (2.00–4.27)
Gender	0.50	0.24	2.1	**0**.**040**	1.65 (1.02–2.65)
Drinking	0.60	0.23	2.6	**0**.**009**	1.82 (1.17–2.85)
Hyperlipidemia	0.88	0.19	4.7	**<**.**001**	2.41 (1.67–3.49)
Smoking	0.47	0.24	2.0	**0**.**045**	1.61 (1.01–2.55)
Diabetes	0.48	0.20	2.4	**0**.**015**	1.62 (1.10–2.39)
Stroke	0.87	0.22	4.1	**<**.**001**	2.39 (1.57–3.65)
Age	0.08	0.01	7.8	**<**.**001**	1.08 (1.06–1.10)
Waistline	0.04	0.01	3.7	**<**.**001**	1.04 (1.02–1.06)

Bold typeface indicates statistically significant differences where the *P* value is less than 0.05.

### Construction and plotting a logistic regression-based predictive model for coronary heart disease risk nomogram

**Figure 1:**
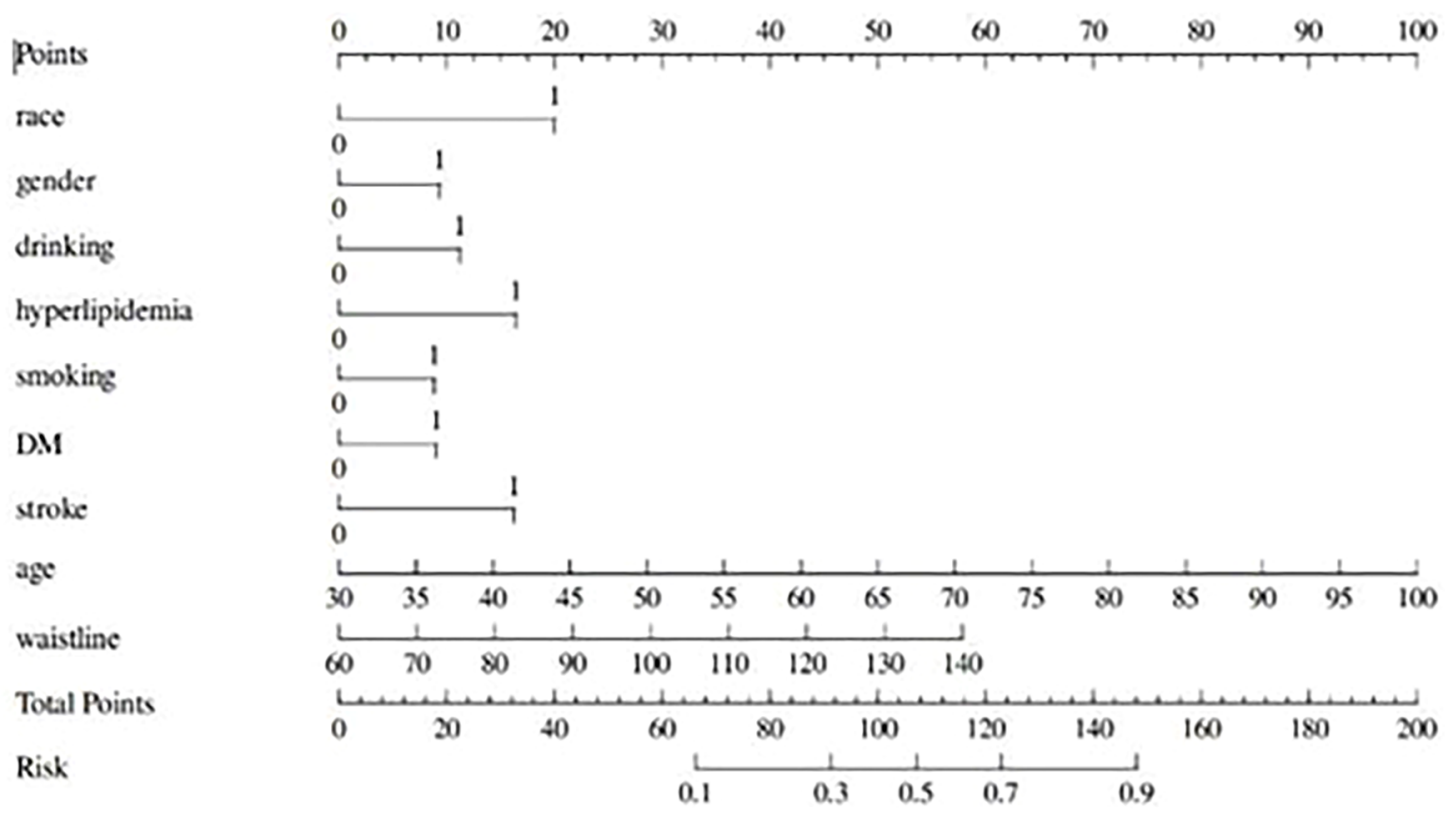
nomogram prediction model for the risk of coronary heart disease. The nine risk factors correspond to specific scores on the nomogram's scale segments, and the individual scores are summed to obtain total points, reflecting the predicted lowest CHD risk of the model. Higher total points indicate a greater risk of CHD.

Based on the nine independent predictors ultimately screened by multifactorial logistic stepwise regression: non-Han ethnicity, male, alcohol consumption, hyperlipidemia, smoking, diabetes mellitus, stroke, age, and waist circumference, the above factors were used to construct and draw the prediction model for the CHD risk nomogram. As shown in Figure 1, the nine risk factors individually correspond to specific scores on the scale segments of the nomogram, and the individual scores for all predictors are summed to yield total points, corresponding to the predicted value of the predictive model's lowest CHD risk. The higher the total points, the higher the risk of CHD.

### Validation and evaluation of predictive models

**Figure 2:**
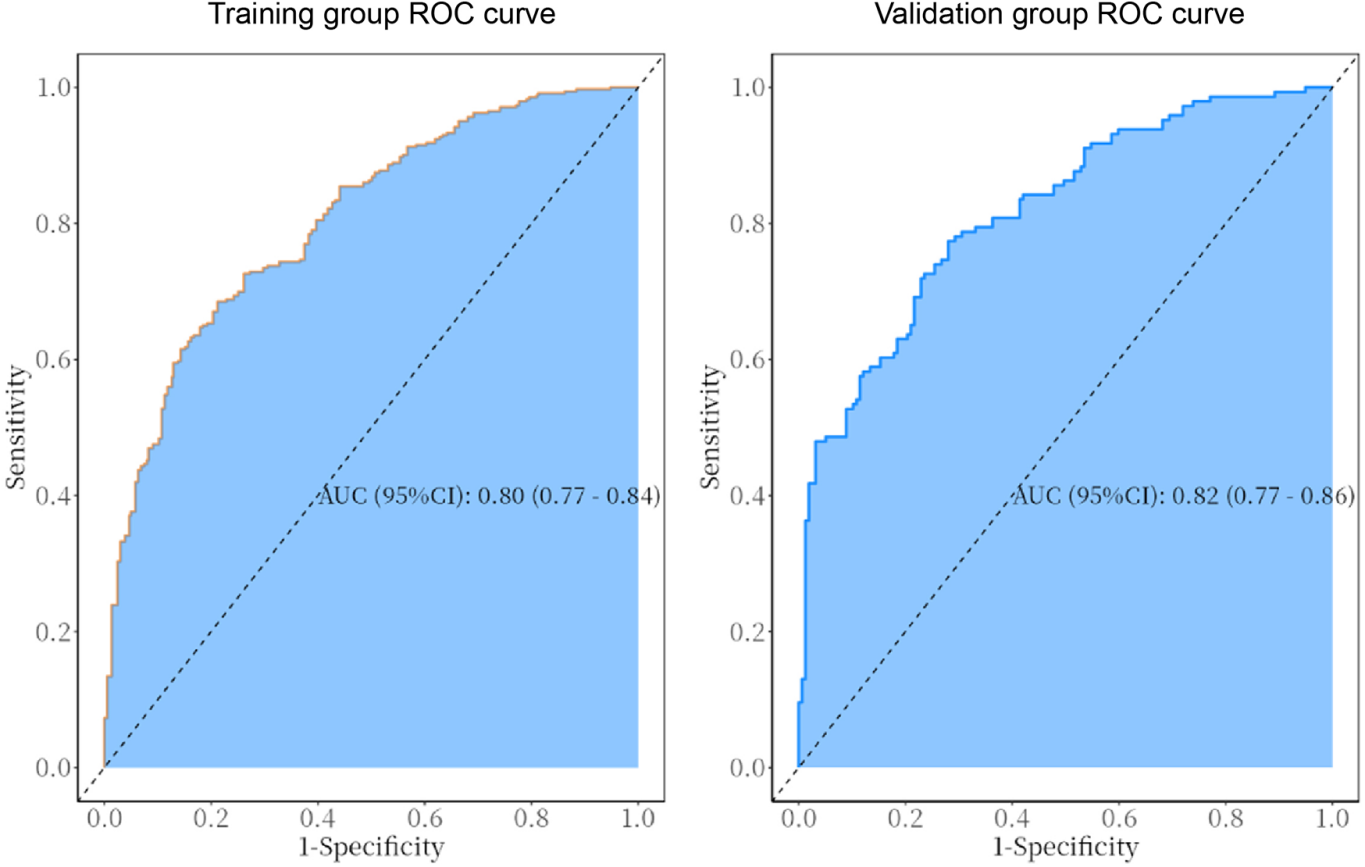
ROC curves of the predictive model in the training and validation group. The AUC for the training group is 0.80 (95% CI: 0.77–0.84), while that for the validation group is 0.82 (95% CI: 0.77–0.86).

**Figure 3:**
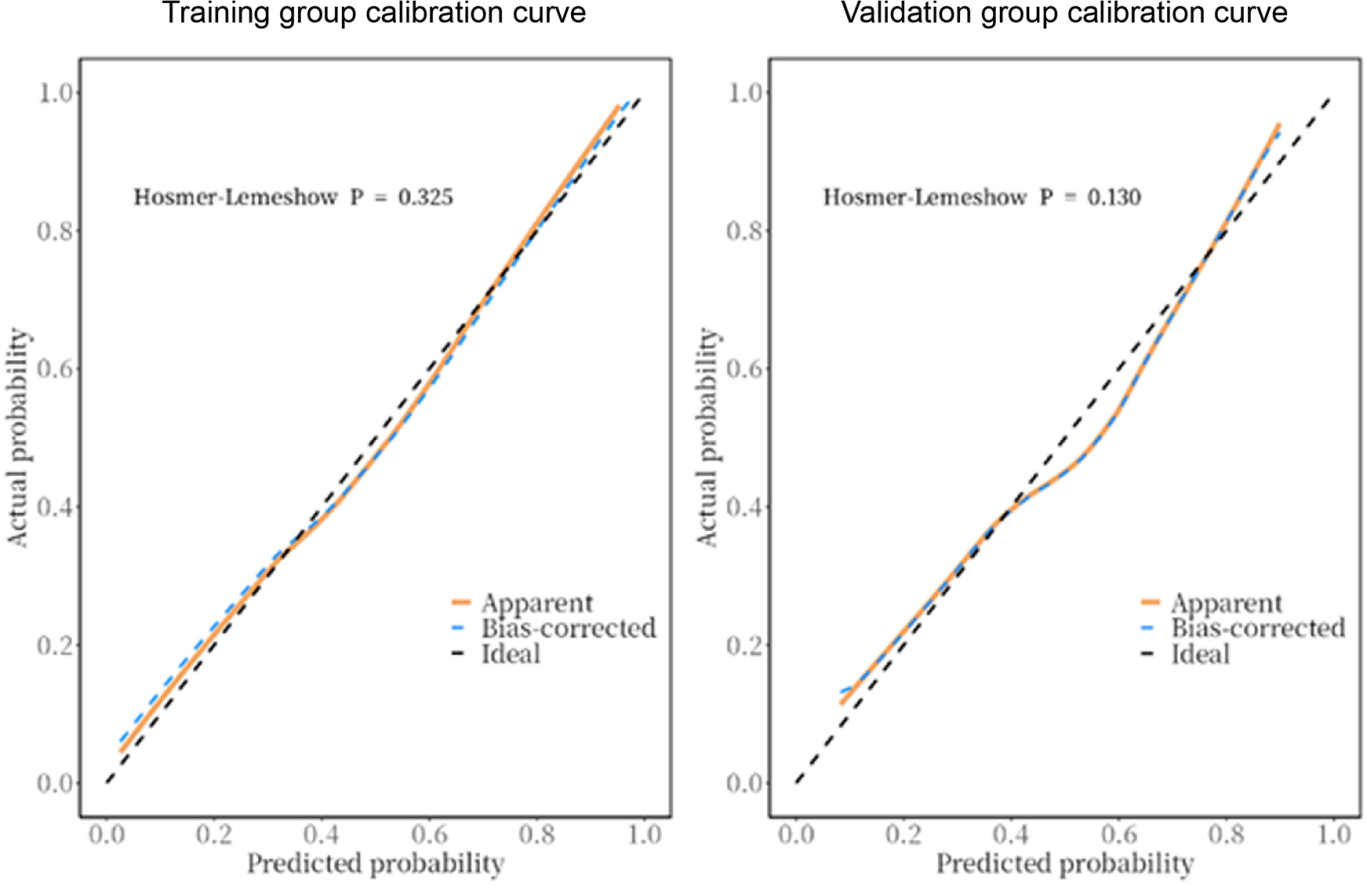
Calibration curves of the prediction model in the training and validation groups. The calibration curve closely matched the ideal curve. The Hosmer-Lemeshow test showed no significant difference between the predicted and actual values for the training group (*P* = 0.325) and validation group (*P* = 0.130), indicating that model predictions were accurate and well-fitted.

**Figure 4:**
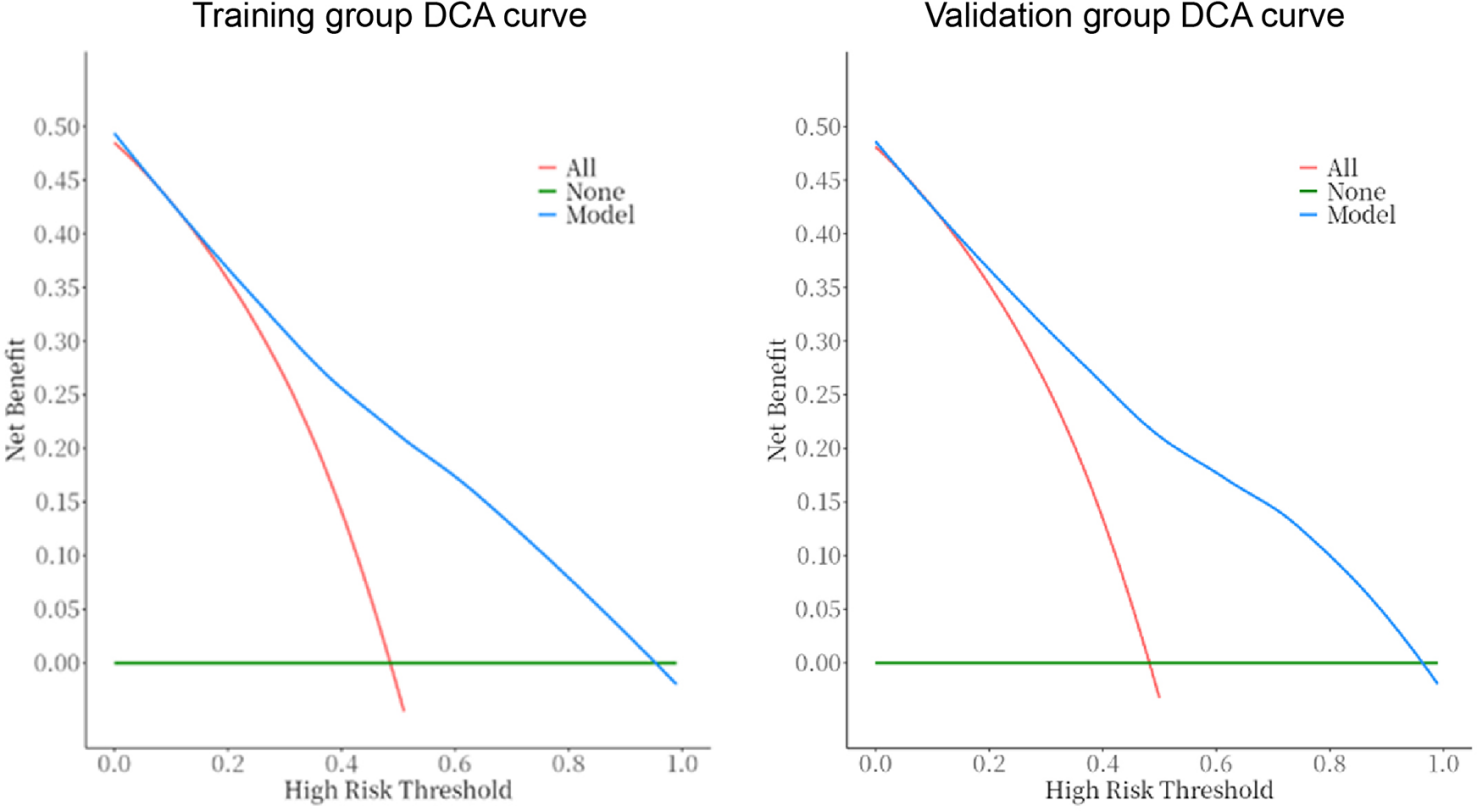
DCA curves of the prediction model in the training and validation groups. The DCA curves for both the training and validation groups indicate that the CHD risk prediction model offers clinical benefits.

The predictive model was applied to the validation group (303 cases) to carry out internal validation. As shown in Figure 2, the AUC of the training group is 0.80 (95% CI: 0.8–0.8), and the AUC of the validation group is 0.82 (95% CI: 0.8–0.9). As shown in Figure 3, the calibration curve did not deviate significantly from the ideal curve. The difference between the predicted value and the actual value of the Hosmer—Lemeshow test for the training group (*P* = 0.325) and the validation group (*P* = 0.130) were not statistically significant (*P* > 0.05), which indicated that the model predictions were close to the true, and the goodness of fit. The results of this study suggest that the model showed good discrimination and calibration in both the training and validation groups, and the prediction model performed well. As shown in Figure 4, the DCA curves in both the training and validation groups suggest that the CHD risk prediction model has clinical benefits.

## Discussion

The findings of this study indicate that non-Han ethnicity, male gender, alcohol consumption, hyperlipidemia, smoking, diabetes mellitus, a stroke, advanced age, and increased waist circumference are significant risk factors for the development of CHD among patients at Tacheng Regional General Hospital in Xinjiang. These factors were found to be positively correlated with the incidence of CHD and have been incorporated into the risk prediction model.

(1) Non-Han: Some studies have found (16) that ethnic minority groups have more risk factors for CHD and are more likely to develop cardiovascular disease, consistent with the results of this study. Several studies have shown that the prevalence of CHD among ethnic minorities such as Kazakhs and Uyghurs in Xinjiang is higher than the national average (16, 17). The Kazakhs and Uyghurs are the major ethnic minorities in the Tacheng area, with different dietary structures, customs, and lifestyles. They prefer foods with high-fat content, cured meats, and milk tea, making them more susceptible to CHD (18).
(2) Male: The PURE study was the first to compare cardiovascular disease differences between genders in different income countries and found that males had a higher cardiovascular risk than females (19), which is consistent with the results of this study. Several studies (20–22) have found that males are more susceptible to CHD risk factors such as abdominal obesity, type 2 diabetes, and hypertension and that there are gender differences in fat distribution, with specific fat distributions in males being more likely to develop CHD. A related study (23) found that males were more likely to suffer from dyslipidemia compared to females due to differences in sex chromosomes and hormone distribution. Studies have shown (24, 25) that men have more unhealthy behaviors and unhealthy dietary patterns and are exposed to more chronic stress. It can be seen that males have more risk factors for CHD, and health-related behaviors and psychosocial factors are different from those of females, leading to a higher incidence of CHD in males.
(3) Alcohol consumption: Some studies (26)have shown that light to moderate drinkers have a lower risk of developing CHD. Current, more mature studies show a J-shaped effect of average alcohol consumption on CHD, with heavy drinkers having the highest risk of CHD (27). The results of this study show that alcohol consumption is an independent risk factor for CHD. Related studies (28–30) have found that alcohol consumption can lead to a significant increase in the risk of hyperlipidemia, hypertension, and diabetes mellitus, all of which are major risk factors for CHD, thereby exacerbating the development of coronary atherosclerosis and leading to an increase in the incidence of CHD. The real reason for the discrepancy in the correlation between alcohol consumption and cardiovascular disease may be that there are uncontrolled risk factors in the study, including mental health, socioeconomic status, social network, and emotional support (31).
(4) Hyperlipidemia: Several studies have shown that hyperlipidemia is an independent risk factor for CHD (32, 33), especially high low-density lipoprotein cholesterol (34). Atherosclerosis begins with the accumulation of low-density lipoprotein cholesterol in the vasculature, followed by endothelial cell activation and dysfunction, inflammation, proliferation, necrosis, and calcification of the vascular wall, and the formation of atherosclerotic plaques (35), which eventually progresses to CHD.
(5) Smoking: A number of studies have confirmed that smoking is a major risk factor for the development of CHD (36, 37). Nicotine, carbon monoxide, and other harmful components in tobacco make the function of vascular endothelial cells impaired, thereby increasing platelet aggregation and adhesion, promoting intimal hyperplasia, accelerating the development of atherosclerotic plaque while smoking sympathetic nerve activity increases significantly, the vascular endothelial cell contraction and relaxation of vascular factor imbalance, resulting in coronary artery spasm and narrowing of the lumen, the resistance to blood flow increases significantly, and further elevate blood viscosity, accelerating the progression of coronary atherosclerosis caused by CHD (38).
(6) Diabetes mellitus: The results of several studies (39, 40) suggest that diabetes mellitus is an independent risk factor for CHD. It has been found (41, 42) that the biochemical reactions in diabetic patients, especially glucose and lipid metabolism, are significantly different from those of healthy individuals. At the same time, insulin resistance exists in their bodies. These complex factors interact to exacerbate endothelial oxidative stress, which induces inflammation and contractile spasm and accelerates the progression of atherosclerosis.
(7) Stroke: Stroke and CHD are both atherosclerotic cardiovascular diseases with common etiologic mechanisms and risk factors (43, 44) and a multicenter study on stroke by Zhang Yan et al. also found that stroke was closely related to CHD or history of angina attacks, etc (45)., which was mutually verified with the results of this paper.
(8) older age: The U.S. Cardiovascular Health Research Institute states that the incidence of coronary heart disease increases with age, and therefore, age is positively correlated with the incidence of CHD (46). Studies related to the age of onset of CHD in the Chinese population have reached the same conclusion (47). With aging, the load on the inner wall of the arterial vessels increases in the elderly, with endothelial damage, increased lipid content within the arterial wall, and increased incidence of atherosclerosis (48), and relevant studies in China have found that the degree of atherosclerosis continues to worsen with age (49), and that severe coronary atherosclerosis progresses to CHD.
(9) Waistline: Various studies (50, 51) support a significant association between waistline and CHD, with an increase in the incidence of CHD as waistline values increase, especially in the young population. The waist has a high content of adipose tissue, especially the accumulation of visceral and subcutaneous fat, and an excessive waistline implies an increase in fat deposition in the abdomen and peripheral organs and the increase and remodeling of adipose tissue, which produces a variety of inflammatory factors, causing vascular dysfunction and the development of cardiovascular disease (52). Increased adipose tissue in the waist can cause a chronic low-grade inflammatory response that affects the endocrine system, triggering metabolic disorders, insulin resistance, dyslipidemia, and hypertension (53, 54). In addition, individuals with excessively large waistlines are often accompanied by poor lifestyle habits, such as lack of exercise, high-calorie diets, and alcohol abuse, which cause metabolic disorders leading to an increase in oxidative stress, accelerating the process of atherosclerosis (55) and increasing the risk of CHD.

## Limitations

Our study has several limitations. First, it was a single-center investigation with a limited sample size; future research will involve multicenter and larger-scale studies to enhance the reliability and generalizability of the prediction model. Second, as the study was conducted in a hospital setting, there may be selection bias among participants, along with constraints on risk factor collection and potential recall bias in clinical data acquisition. Furthermore, we did not adjust for medications such as lipid-lowering agents, antihypertensives, and antidiabetic drugs due to insufficient data availability; this oversight may have influenced our findings. Lastly, external validation of our model is lacking. Future studies should aim to increase sample sizes, incorporate additional risk factors, and perform external validation to bolster the model's applicability.

## Conclusions

This study developed a risk prediction model for CHD in Tacheng. Validation results showed AUC values of 0.80 and 0.82 for the training and validation sets, respectively, indicating high discriminatory ability. The Hosmer-Lemeshow test and calibration curve confirmed that the model fit well and demonstrated strong predictive performance. Additionally, decision curve analysis indicated that this model offers net clinical benefits and effectively assesses CHD risk. The nomogram provides an objective way to evaluate risk and identify high-risk individuals, facilitating early intervention and supporting CHD prevention efforts in Tacheng.

## Data Availability

The original contributions presented in the study are included in the article/Supplementary Material, further inquiries can be directed to the corresponding author.
